# The metabolites NADP^+^ and NADPH are the targets of the circadian protein Nocturnin (Curled)

**DOI:** 10.1038/s41467-019-10125-z

**Published:** 2019-05-30

**Authors:** Michael A. Estrella, Jin Du, Li Chen, Sneha Rath, Eliza Prangley, Alisha Chitrakar, Tsutomu Aoki, Paul Schedl, Joshua Rabinowitz, Alexei Korennykh

**Affiliations:** 1 216 Schultz Laboratory, Department of Molecular Biology, Princeton, NJ 08544 USA; 2 285 Frick Laboratory, Department of Chemistry, Princeton, NJ 08544 USA; 3 Lewis-Sigler Institute for Integrative Genomics, Princeton, NJ 08544 USA

**Keywords:** Structural biology, Cell biology, Biochemistry, Molecular biology

## Abstract

Nocturnin (NOCT) is a rhythmically expressed protein that regulates metabolism under the control of circadian clock. It has been proposed that NOCT deadenylates and regulates metabolic enzyme mRNAs. However, in contrast to other deadenylases, purified NOCT lacks the deadenylase activity. To identify the substrate of NOCT, we conducted a mass spectrometry screen and report that NOCT specifically and directly converts the dinucleotide NADP^+^ into NAD^+^ and NADPH into NADH. Further, we demonstrate that the *Drosophila* NOCT ortholog, Curled, has the same enzymatic activity. We obtained the 2.7 Å crystal structure of the human NOCT**•**NADPH complex, which revealed that NOCT recognizes the chemically unique ribose-phosphate backbone of the metabolite, placing the 2′-terminal phosphate productively for removal. We provide evidence for NOCT targeting to mitochondria and propose that NADP(H) regulation, which takes place at least in part in mitochondria, establishes the molecular link between circadian clock and metabolism.

## Abstract (web summary)

Nocturnin is a rhythmically expressed protein that regulates metabolism under the control of circadian clock proposed to function through the deadenylation of metabolic enzyme mRNAs. Here the authors show that Nocturnin and its fly homolog Curled catalyze the removal of 2′-phosphate from NADP^+^ and NADPH, providing a direct link to metabolic regulation.

## Introduction

The circadian clock adjusts metabolism and behavior of living organisms according to day and night periodicity^1,2^. A screen for circadian genes using *Xenopus laevis* retinas identified the ~50 kDa protein Nocturnin (NOCT), which exhibited a rhythmic expression that peaks at night (16 h Zeitgeber time, ZT16)^3,4^. The emergence of sequenced genomes revealed that NOCT is conserved from flies to humans^5–7^. It has been determined that the first description of the NOCT gene happened more than a hundred years ago during studies of *Drosophila melanogaster* (fruit fly) by Thomas Hunt Morgan. The fruit fly NOCT ortholog was called Curled (*cu*) due to a peculiar upward wing curvature in *cu* flies^5^. The *cu* mutant has become a widely used marker in fruit fly genetics and the curled phenotype has been linked to defects in metabolism^5^. In mice, NOCT mRNA undergoes a high-amplitude regulation by circadian clock, peaking by two orders of magnitude in the liver in the transition to evening (ZT12)^8^. NOCT^−/−^ mice exhibit altered lipid metabolism on a high-fat diet and do not develop fatty liver disease (hepatic steatosis) or obesity that occur in WT mice^6^. The NOCT^−/−^ mice have low sensitivity to insulin and glucose, and have altered nitric oxide signaling, indicating a deep integration of this circadian protein in mammalian metabolism^6,9–11^.

NOCT is a member of the exonuclease/endonuclease/phosphatase family of proteins which include PDE12, a mitochondrial deadenylase needed for maturation of mitochondrial tRNAs, and CNOT6L, the mammalian ortholog of the main cytosolic yeast deadenylase CCR4^12,13^. Crude NOCT preparations were reported to deadenylate mRNA in vitro, leading to the model that NOCT is a circadian deadenylase acting on mRNAs^4^. Regulation of insulin sensing, nitric oxide signaling, and lipid metabolism was therefore attributed to a globally altered stability of metabolic enzyme mRNAs^6,9,11^. As a growing body of literature linked the biological effects of NOCT to mRNA deadenylation, two reports described the lack of deadenylase activity in highly purified NOCT in vitro^7,14^. Under the same experimental conditions, PDE12 and CNOT6L were active and readily degraded poly-A RNA^7,14^.

To understand the lack of poly-A RNA cleavage by human NOCT, both groups determined the crystal structure of its catalytic domain. The structural studies could not explain the paradoxical inactivity of NOCT, revealing the same fold and the catalytic center as in PDE12 and CNOT6L^7,14^. It has been proposed that NOCT may require a protein partner perhaps for recruitment to mRNAs. Alternatively, it has been postulated that NOCT may have a substrate distinct from poly-A RNA. Here we confirm the second model and show direct cleavage of two chemically related metabolites by human NOCT and by its fruit fly ortholog, Curled.

## Results

### NADP^+^ and NADPH are the direct substrates of NOCT

**Fig. 1:**
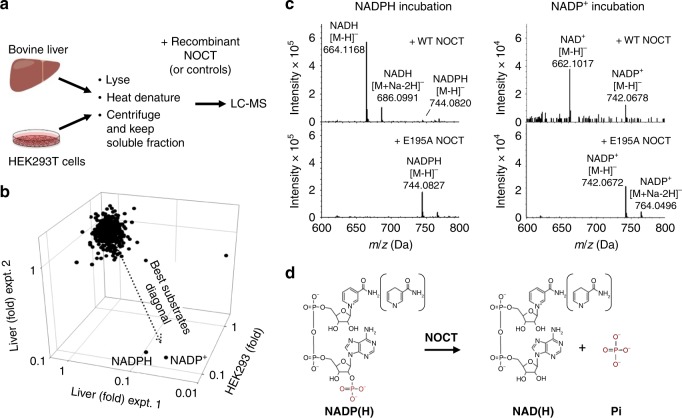
Metabolite profiling to identify NOCT substrate. **a** Metabolite extraction and treatment outline. Full-length human NOCT and the catalytic domain of NOCT (residues 122–431) were compared against full-length human NOCT E195A, NOCT (122–431) E195A mutant, or the catalytic domain of CNOT6L (158–555). Liver experiment 1 and HEK293 experiment used the catalytic domain of NOCT. Liver experiment 2 used GST-tagged full-length NOCT immobilized on glutathione beads. **b** Metabolite depletion in three independent experiments (Supplementary Data Set 1). Fold is defined as the ratio of LC-MS intensities for samples with WT NOCT vs average for samples with NOCT E195A and CNOT6L. **c** LC-MS/MS analysis of purified NADP^+^ and NADPH following incubation with WT or E195A human NOCT (122–431; 10 μM, 1 h at r.t.). **d** The identified reactions catalyzed by human NOCT

**Fig. 2:**
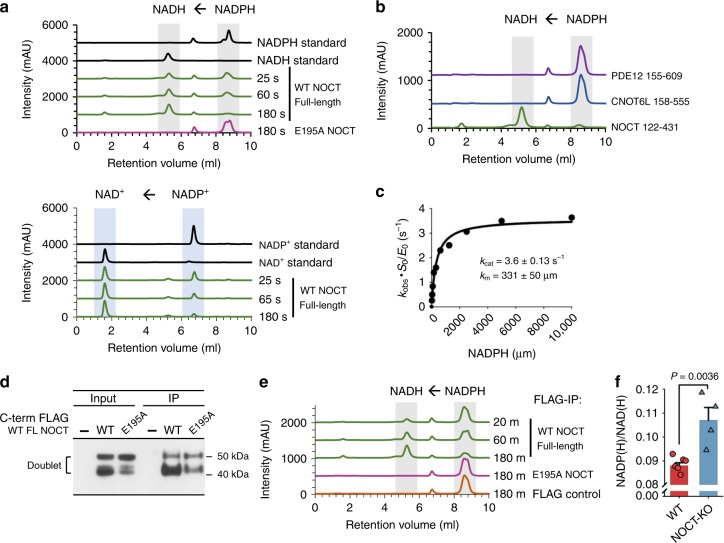
NADP(H) cleavage characterization. **a** Time course cleavage of pure NADPH (1 mM) and NADP^+^ (1 mM) by purified human full-length NOCT (0.5 μM) at 22 °C, monitored by ion-exchange chromatography (IEC). **b** 60-min NADPH incubation with the catalytic domains of PDE12, CNOT6L, and NOCT as in **a**. **c** Measurement of kinetic parameters for the catalytic domain of human NOCT (4.8 μM). **d** Western blot analysis of full-length human FLAG-NOCT (C-terminal tag) expressed in A549 cells for 18 h. **e** NADPH (1 mM) cleavage by FLAG-NOCT purified from human cells. **f** Nicotinamide metabolite composition in WT and NOCT^−/−^ cells (Supplementary Data 2). Six replicates from three WT samples are compared against four replicates from two independently generated NOCT^−/−^ clones. Error bars are standard error (S.E.). Source data are provided as a Source Data file

Based on the absence of mRNA deadenylase activity in purified NOCT, and its known role in metabolism, we hypothesized that NOCT could cleave a metabolite. A previously reported candidate-based approach was unable to find the NOCT substrate^14^, therefore we used an unbiased screen based on metabolomics mass spectrometry. We extracted metabolites from a freshly obtained bovine liver, and independently, from human embryonic kidney HEK293T cells (Fig. 1a). We treated the metabolite extracts with the catalytic domain of recombinant human NOCT (Supplementary Fig. 1a). NOCT E195A, a point mutant disrupting magnesium coordination^7,^ and WT human deadenylase CNOT6L (residues 158–555) were used as negative controls. Liquid chromatography-mass spectrometry analysis (LC-MS) of the samples revealed that two related metabolites, NADP^+^ and NADPH were selectively depleted by WT NOCT (Fig. 1b; Supplementary Data 1). Their depletion coincided with enrichment in NAD^+^ and NADH (Supplementary Fig. 1b). These results were reproduced using pure commercially available NADP^+^ and NADPH: the addition of WT NOCT catalytic domain, but not the E195A mutant converted NADPH into NADH and NADP^+^ into NAD^+^ (Fig. 1c). Thus, NOCT uses NADP^+^ and NADPH as substrates and removes the terminal 2′-phosphate (Fig. 1d). To facilitate further analyses of the NADP(H) cleavage reaction, we substituted LC-MS with ion-exchange chromatography (IEC). Individual nicotinamide metabolites could be resolved by this technique and quantified by UV absorbance at 260 nm. The IEC assay showed a time-dependent quantitative conversion of both NADP^+^ and NADPH into NAD^+^ and NADH, respectively by full-length (FL) NOCT (Fig. 2a). The activity was uniquely present in NOCT, both full-length and catalytic domain, and absent in the control reactions with human deadenylases CNOT6L and PDE12 (Fig. 2b).

Using conditions of kinetic competition that are established by an equimolar mixture of NADP^+^ and NADPH, we determined that NOCT has a small preference for NADPH (Supplementary Fig. 2a). A titration experiment revealed that NOCT cleaves NADPH with *K*_m_ of approximately 170–300 μM and the first-order catalytic constant *k*_cat_ = 3.6 s^−1^ for the catalytic domain (Fig. 2c) and 34 s^−1^ for the full-length protein (Supplementary Fig. 2c). The *K*_m_ is comparable to that of other enzymes involved in NADP(H) metabolism. The *K*_m_ of NOCT is within 2-3-fold from that of the recently identified cytosolic NADP(H) phosphatase MESH1 (*K*_m_ = 0.11 mM)^15^. Further, the NOCT *K*_m_ is about 5–10-fold better than that for the kinases NADK and NADK2 that synthesize NADPH from NADH (1.7–3.6 mM)^16^. The phosphatase activity observed in NOCT purified from a bacterial expression system was reproduced with overexpressed full-length human NOCT purified from human cells (Fig. 2d, e; Supplementary Fig. 2b).

To probe regulation of cellular NADP(H) by NOCT, we generated NOCT-KO A549 human cell lines and purified metabolites to determine the NADP(H)/NAD(H) ratios. Faithful capture and quantitation of nicotinamide metabolites in subcellular organelles remain challenging, therefore we carried out whole-cell LC-MS. Analysis of the total cellular metabolites showed a modest, but significant whole-cell increase for NADP^+^ and NADPH in NOCT-KO cells, compared to the 2′-dephosphorylated NAD forms (Fig. 2f; Supplementary Data 2). This experiment may be underestimating the magnitude of 2′-phospho NAD changes inside individual organelles.

### NOCT is targeted to mitochondria

**Fig. 3:**
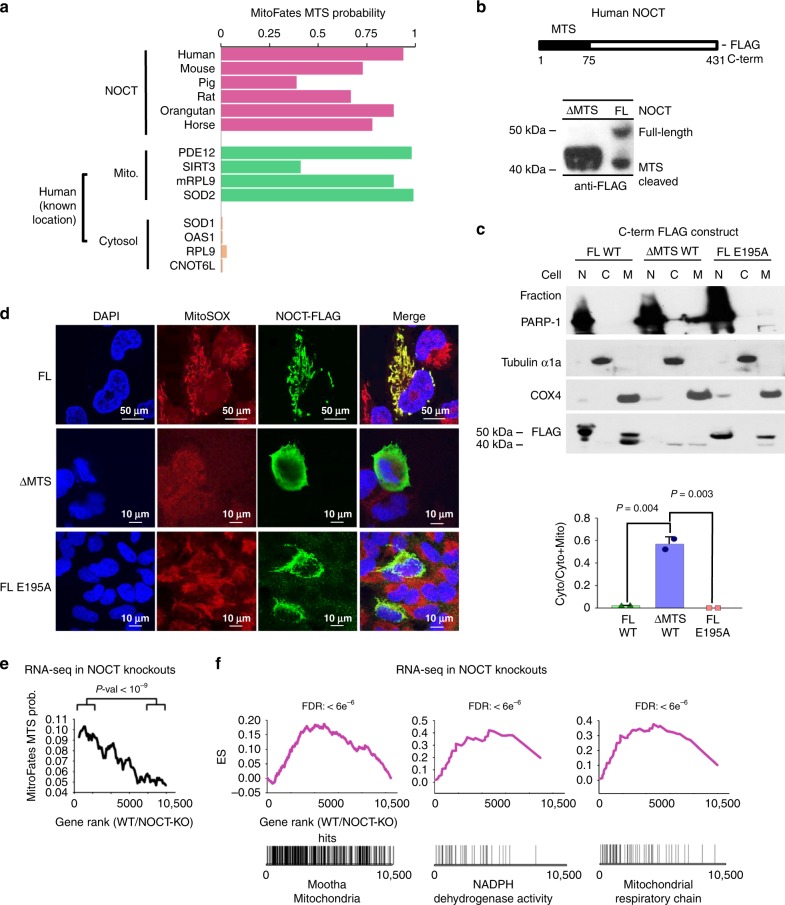
Evidence for NOCT mitochondrial localization. **a** MitoFates analysis of NOCT and reference human proteins. **b** Western blot for FLAG-NOCT, full-length vs ΔMTS. **c** Subcellular fractionation and western blot analysis of FLAG-NOCT, WT, ΔMTS, and E195A active site mutant. Error bars show S.E. from the data in **c** and Supplementary Fig. 3a, *P*-values here and throughout were determined by Welch’s two-tailed unpaired *t*-test. PARP-1, Tubulin, and COX4 mark the nucleus, the cytosol, and the mitochondria, respectively. **d** Confocal microscopy of NOCT variants in **c**. DAPI stains for nuclei, MitoSOX stains mitochondria. **e** Running average for MitoFates scores for ~10,500 mRNAs ordered according to gene expression levels in WT vs NOCT^−/−^ cells, measured by RNA-seq (data are provided in Supplementary Data 3). *P*-values for top 2000 vs bottom 2000 mRNAs. Error bars represent S.E. RNA-seq data represent four RNA-seq experiments: two in WT cells and two in independently derived NOCT-KO clones. **f** GSEA enrichment results for the genes with ≥50 reads, ranked according to WT/NOCT^−/−^ expression as in **e** (Supplementary Data 3). Source data are provided as a Source Data file

During expression of NOCT in human cells, we noticed that the protein migrated as two discrete bands, which is indicative of cellular processing (Fig. 2d). To understand this observation, we used bioinformatics and found that NOCT has a predicted mitochondrial targeting sequence^17^ (MTS, Fig. 3a). The same analysis can discern the presence of an MTS or lack thereof in known mitochondria-targeted proteins or cytosolic proteins, respectively. To test whether the MTS of human NOCT is functional, we generated the deletion mutant, ΔMTS-NOCT. This variant migrated as one band corresponding to the size of processed NOCT (Fig. 3b). Using subcellular fractionation, we detected NOCT with processed MTS predominantly in the mitochondrial fraction (Fig. 3c; Supplementary Fig. 3a). Unprocessed NOCT was present partially in mitochondria and partially in the nucleus (Fig. 3c; Supplementary Fig. 3a), localizing to the nucleus likely as a consequence of overexpression. Using confocal microscopy, we found that full-length NOCT was targeted distinctly to mitochondria, whereas ΔMTS-NOCT localized to the cytosol, as expected (Fig. 3d; Supplementary Fig. 3b).

To probe the cellular effects of NOCT, we analyzed gene expression differences in WT vs NOCT-KO human cells by RNA-seq (Supplementary Data 3). Compared to NOCT-KO cells, WT cells had increased levels of mRNAs encoding proteins with high-confidence mitochondrial localization based on MTS motifs^17^ and MitoCarta 2 scores^18^ (Fig. 3e; Supplementary Fig. 3c). In agreement with these data, multiple hypotheses testing analysis in GSEA^19^ found gene set enrichments with false discovery rates (FDR) < 0.001 for mRNAs encoding proteins involved in mitochondrial organization and mitochondrial processes (Fig. 3f and Supplementary Fig. 3d). Considering that NOCT cleaves NADP(H) rather than mRNAs, the upregulation of multiple mRNAs in the presence of NOCT is no longer surprising and can be explained by a response to NADP(H) modulation. Together, these RNA-seq results and the presence of an MTS in NOCT point to an important mitochondrial function of NOCT.

### Structure of the complex between human NOCT and NADPH

**Fig. 4:**
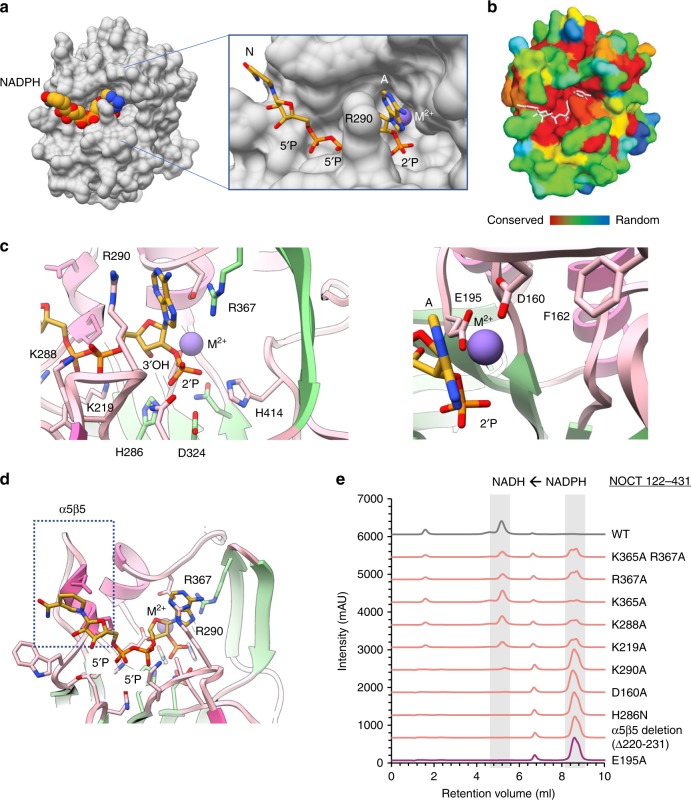
Structure of human NOCT•NADPH•Ca^2+^ complex. **a** Surface representation of the complex with NADPH bound to the active site pocket. **b** NOCT conservation from 351 non-degenerate sequences mapped onto the NOCT structure. Color scale shows a range from invariable (red) to random (blue). **c** NADPH position and the divalent metal ion coordination in the catalytic center. **d** NADPH position relative to the α5β5 motif. **e** NADPH cleavage by NOCT mutants designed to disrupt the enzymatic activity. NOCT catalytic domain (500 nM) and NADPH (0.5 mM) were used. Reactions were conducted at 22 °C for 60 min

To understand precisely how NOCT recognizes NADPH, we obtained co-crystals by using calcium instead of magnesium to inhibit the activity of WT NOCT (Supplementary Fig. 4a) and to stabilize the NOCT**•**NADPH complex. We determined the 2.7 Å crystal structure of the NOCT**•**NADPH complex (Supplementary Table 1; Supplementary Fig. 4b) and found the metabolite bound in a deep pocket comprised of residues that are conserved in the NOCT family^7^ (Fig. 4a, b; Supplementary Fig. 4c, d). NOCT forms few contacts with the nucleobases. The nicotinamide group does not pack strongly against any NOCT residues and has weak electron density. The adenine is sandwiched between two arginines (R290 and R367) via cation-pi interactions. The density for the adenine is relatively weak, indicating that these interactions are relatively dynamic (Fig. 4c; Supplementary Fig. 4d). In contrast, the ribose-5′-PP-5′-ribose-2′-P backbone of NADPH forms multiple stable contacts and has better electron density. The recognition of the sugar-phosphate backbone appears to be incompatible with other cellular ligands, including ATP, RNAs, mRNA 5′-cap (which has ribose-5′-PPP-5′-ribose linkage, and 3′-P rather than 2′-P at the scissile position), and NAD^+^-capped mRNAs^20^ containing 3′-P rather than 2′-P at the scissile position.

The 2′-P of NADPH is placed in a chemically ideal position for dephosphorylation (Fig. 4c). The chemical step for the ribose-phosphate hydrolysis should be the same as in the related mRNA deadenylases PDE12 and CNOT6L, and the DNA repair enzyme TDP2 due to superimposition of their magnesium-coordinating catalytic centers^7,13,21^. For TDP2, which has been crystallized with DNA, the scissile 5′-phosphate and the catalytic Mg^2+^ ion were positioned with confidence in the active site aided by the size of the DNA substrate. Superimposition of our NOCT**•**Ca^2+^**•**NADPH structure with this TDP2**•**Mg^2+^**•**DNA complex shows identical metal ion and phosphate placements, indicates that the phosphatase activity of NOCT and the phosphodiesterase activity of TDP2 indeed involve the same chemistry (Supplementary Fig. 4e, f). In NOCT, the phosphate coordinates with the catalytic metal ion, which is positioned to stabilize the leaving group (ribose oxygen). The 3′-hydroxyl is buried inside the active site and away from the metal ion. The orientation of the ribose near the catalytic metal ion places the 2′ ribose position rather than the 3′ ribose position for chemical attack. This can explain the inability of NOCT to cleave RNA. The catalytic water molecule that serves as the incoming nucleophile must be activated by ASP324 (Fig. 4d), which is the only residue with pK_a_ near neutrality next to the nucleophile position. The mode of NADPH recognition by NOCT is unique and bears little resemblance with the only other professional NADP(H) phosphatase described to date, MESH1 (PDB ID 5VXA)^15^. In MESH1 nucleobases rather than the sugar-phosphate backbone form the majority of protein-substrate contacts, whereas the catalytic metal ion Zn^2+^ is placed to activate the nucleophilic water molecule rather than the leaving 2′-O group of the ribose.

Bioinformatics analysis has shown that the helix α5 linked to the strand β5 (α5β5 motif) is a conserved structural feature of NOCT that is not conserved in homologous mRNA deadenylases^7^ (Fig. 4d). In the NOCT**•**NADPH complex, the α5β5 motif packs closely with the nicotinamide-bearing sugar. Superimposing structures illustrates that neither CNOT6L nor PDE12 has the α5β5 motif in the position for binding NADPH (Supplementary Fig. S4G). Additionally, the mRNA deadenylases contain a tryptophan instead of an alanine present in NOCT (Supplementary Fig. 4h), which creates a steric clash with the diphosphate-sugar backbone of NADPH and further distinguishes NOCT from mRNA deadenylases. We mutated a number of NOCT active site residues proximal to NADPH, as well as deleted the α5β5 motif and determined that all of these mutations are detrimental to the NADPH 2′-dephosphorylation activity (Fig. 4e).

### *Drosophila Melanogaster* Curled cleaves NADP^+^ and NADPH

**Fig. 5:**
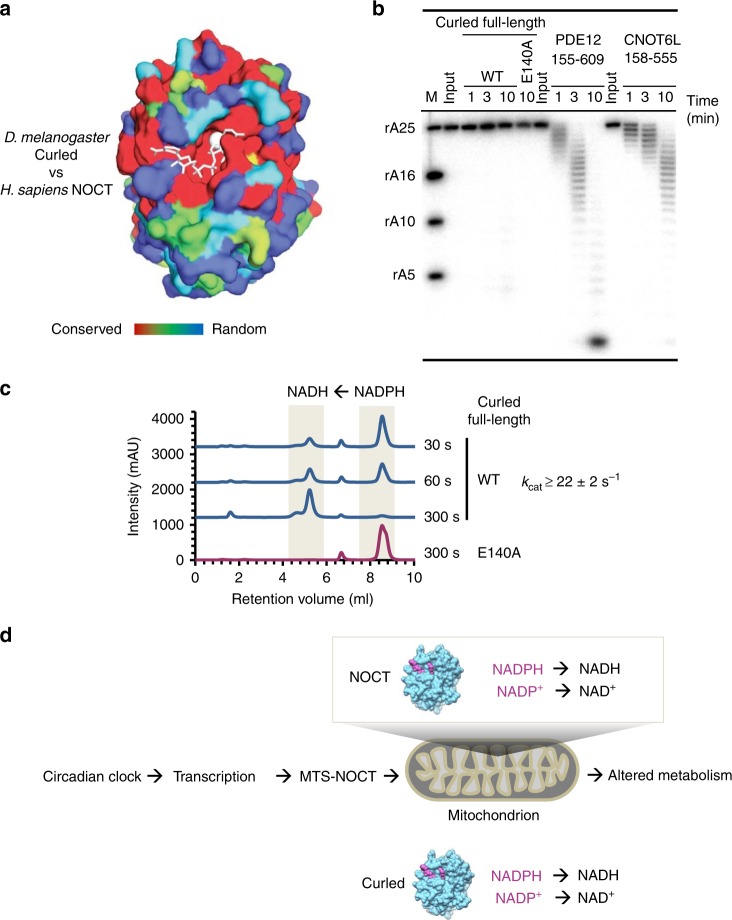
Biochemical characterization of Curled. **a** Structure of human NOCT colored by sequence conservation between fruit fly and human proteins. Color scale shows a range from invariable (red) to random (blue). **b** ^32^P-poly-A RNA cleavage assays using *D. melanogaster* Curled, Curled E140A active site mutant, and human deadenylases CNOT6L and PDE12 (2 μM each). Faint bands at 10 min in WT Curled arise from a minor contaminating endonuclease. The E140A mutant corresponds to the E195A mutation in human NOCT (Figs. 2a and 4e). **c** IEC trace for cleavage of NADPH (1 mM) by Curled from *Drosophila Melanogaster* (500 nM). The catalytic constant was calculated as *k*_obs_**•**S_0_/E_0_ from (**c**) and Supplementary Fig. 5b. This value represents the lower estimate for *k*_cat_. **d** The proposed model for NOCT integration in the circadian system based on the results of our work. Source data are provided as a Source Data file

Conservation analysis shows that the NADPH-binding pocket is conserved between human NOCT and Curled, suggesting that our findings with human NOCT may extend to the fly ortholog (Fig. 5a). To test this prediction, we cloned and purified the full-length *Drosophila melanogaster* protein (Supplementary Fig. 5a) and tested its RNase and 2′-phosphatase activities. We determined that Curled lacks deadenylase activity when tested using radiolabeled poly-A RNA. Neither WT Curled nor Curled E140A active site mutant cleaved poly-A, whereas the control deadenylases CNOT6L and PDE12 degraded the RNA substrate within minutes (Fig. 5b). In contrast, Curled readily cleaved NADPH (Fig. 5c; Supplementary Fig. 5b). This catalytic activity was blocked by the active site point mutant E140A, which mimics the E195A mutation in human NOCT. Further extending the similarity with human NOCT, Curled cleaved both NADP^+^ and NADPH, exhibiting a preference for NADPH (Supplementary Fig. 5c). Full-length Curled shows ~2-fold preference for NADPH (Supplementary Fig. 5c), which is comparable to the 6-fold preference observed with full-length NOCT (Supplementary Fig. 2a). For NADP^+^ the specific cleavage activity of Curled and NOCT are the same, whereas for NADPH NOCT is ~3–4-fold more active.

## Discussion

We identified NADP^+^ and NADPH as the substrates of the circadian enzyme, Nocturnin. Although the involvement of NOCT in metabolism has been widely recognized, it had not been expected that this enzyme directly regulates the central cofactors in anabolic and catabolic reactions, NADP^+^ and NADPH (substrates), and NAD^+^ and NADH (products); NOCT does not degrade NADP(H), but converts it into a coupled  molecule NAD(H) essential for energy metabolism^22^. Enzymes that use NADPH are key to mitochondrial oxidation of unsaturated lipids and oxidative stress management^23^, as well as regulation of body weight and insulin sensing^24,25^, which represent the major phenotypes reported for NOCT. Previously, the physiological effects of NOCT have been attributed to metabolic mRNA deadenylation^11^. Our work shifts the focus to the direct target, NADP(H), and thereby establishes  the grounds for re-interpretation and mechanistic understanding of the functions of this enzyme in metabolism and circadian clock output. For example, the sharp peak in NOCT expression when mice wake up (mice are nocturnal and ZT12 corresponds to awakening) must be understood from the point of NADP(H)/NAD(H) regulation. One physiological function of NOCT could be to maximize available NAD(H) for energy generation in a search for food, using elevated blood sugar that animals have at the time of awakening^26^.

The demonstration that Curled cleaves NADPH reveals the molecular target of the mutant *cu*, which has been used for more than a century in fruit fly genetics. This result bridges a 103-year gap in knowledge and in addition verifies that NADP(H) 2′-dephosphorylation is a conserved activity in the NOCT family. Of note, in 1923, Lenore Ward described a dominant mutation, Curly, which caused upward wing curvature analogous to that seen in Curled. It has been established recently that the Curly gene is not related to Curled and encodes an NADPH oxidase that generates reactive oxygen species (ROS)^27^. Therefore, both genes linked to the curled wing phenotype, Curled and Curly, share the same NADPH substrate, suggesting that they may regulate the same or overlapping pathways controlling wing morphology, and that these activities could depend on ROS signaling.

Human NOCT is encoded by a single isoform with an MTS, which can target the enzyme to mitochondria. At least some of the biological functions of human NOCT, therefore, involve cleavage of mitochondrial NADP(H) (Fig. 5d). The MTS is present in the NOCT gene of higher animals, but in *Drosophila melanogaster* only two of the six isoforms are predicted to have an MTS. These observations suggest that Curled and perhaps also NOCT may have functions inside and outside of mitochondria. In conclusion, we propose that NOCT/Curled convert NADP^+^ to NAD^+^ and NADPH to NADH to regulate metabolism and in the case of NOCT, to synchronize metabolism with the biological clock.

Presently, NOCT has Enzyme Commission (EC) classification 3.1.13.4: poly(A)-specific 3′-exoribonuclease^28^. We show that NOCT does not position the 3′ hydroxyl productively in the active site and that instead NOCT is an NADP(H) 2′-phosphatase, providing an activity that, unexpectedly, does not match any current EC class. At the moment there is only one additional example of a dedicated NADP(H) 2′-phosphatase, MESH1, described in a preprint publication^15^. NOCT has a unique structure and NADPH binding mode, compared to MESH1. Moreover, the association with the circadian clock and fly phenotypes highlights the importance of the NADP(H) phosphatase reaction. Therefore, we call for the creation of an EC entry to describe this enzyme class.

## Methods

### Cloning

The coding region of full-length human Nocturnin (residues 1–431) was amplified by PCR from an in-house cDNA library using poly I:C transfected A549 human lung epithelial cells (obtained from Prof. Susan Weiss, UPenn) to upregulate NOCT expression. The *Drosophila melanogaster* Curled cDNA (splicing form H) was cloned by RT-PCR from the RNA pool of wild-type fly embryos. Seven micrograms of total RNA from Oregon-R embryo was reverse-transcribed by using the primer cu-02 (Supplementary Table 2) followed by the PCR reaction with forward (XhoI-cu-03) and reverse (NotI-cu-06) primers. The single band of PCR product was purified from agarose gel and then subcloned into pCRII vector (Invitrogen). The cDNA clone did not include any base changes that cause amino-acid alteration from Curled protein sequences in the FlyBase (https://flybase.org). The PCR products were cloned into pGEX-6P bacterial expression vector (GE Healthcare Life Sciences). Deletion and point mutants were generated by one-primer site-directed mutagenesis as previously described^7^. The constructs for human PDE12 155–609 and CNOT6L 158–555 were also previously described^7^. For mammalian expression, full-length NOCT, ΔMTS-NOCT (75–431), and full-length NOCT-E195A were subcloned into pcDNA^TM^4/TO vector (Life Technologies). All constructs used in this study were verified by DNA sequencing.

### Protein purification

pGEX-6P vectors encoding recombinant proteins were transformed into *Escherichia coli* BL21 (DE3)-CodonPlus RIPL (Agilent Technologies) and grown to an OD600 of 0.4 in Luria-Bertani medium at 37 °C followed by induction with 0.2 mM isopropyl-β-D-thiogalactopyranoside (IPTG) and overnight expression at 22 °C. The cells were pelleted at 4600 × *g* for 20 min, resuspended in lysis buffer (20 mM HEPES pH 7.4, 1 M KCl, 2 mM MgCl_2_, 1 mM EDTA, 10% (vol/vol) glycerol, 5 mM DTT, and Roche complete protease inhibitor), and lysed on EmulsiFlex C3 (Avestin).

Crude lysates were clarified by centrifugation at 35,000 × *g* for 30 min, at 4 °C. Clarified lysates were affinity-purified using glutathione Sepharose (GE Healthcare Life Sciences). The GST tag was removed with Prescission protease (GE Healthcare Life Sciences). NOCT 122–431, PDE12 155–609, CNOT6L 158–555 and Curled 1–419 variants were similarly purified. Full-length NOCT was further purified by MonoQ, then by MonoS ion-exchange chromatography, and lastly, by Superdex S200 size-exclusion chromatography. NOCT constructs were purified using MonoS, MonoQ, and Superdex 200 size-exclusion chromatography. For PDE12, CNOT6L and Curled only size-exclusion chromatography was used. Size-exclusion buffer contained 20 mM HEPES (pH7.4), 350 mM KCl, 1 mM EDTA, 10% (vol/vol) glycerol, and 5 mM DTT. All proteins were purified to ≥95% purity. Concentrations were determined by UV 280 spectrophotometry, using protein sequence for calculating extinction coefficients.

### Metabolite extraction and LC-MS

Cow liver (purchased fresh from Cherry Grove Farm, Lawrenceville, NJ, USA) was homogenized using Retsch Cyromill and the homogenate (~0.8 g) was resuspended in 600 μl sterile water. For HEK293T cells, nine 10 cm dishes containing confluent cells were washed with 2 ml of cold phosphate-buffered saline (PBS). Cells (~0.4 grams) were scraped off and spun down at 100 × *g* for 5 min in 1.5 ml tube. The supernatant was discarded and the cells were resuspended in 300 μl sterile water. From this point forward liver and HEK293T cells were treated similarly.

The suspensions were sonicated on ice on Branson Sonifier S-450 with 20 pulses (Duty Cycle: 30%, Output: 4). The lysates were heated to 95 °C for 10 min then centrifuged at 22,000 × *g* for 10 min (4 °C). The cleared lysates were used to set up separate reactions with soluble NOCT, E195A NOCT, CNOT6L, and blank in a total volume of 135 μl (liver) or 90 μl (HEK293T). For bead-immobilized full-length NOCT, 100 μl of bed volume was used per reaction. Each reaction tube was supplemented with 10 μM proteins (f/c) (except blank and bead samples), 0.5 mM MgCl_2_, and incubated for 19 h at 22 °C. The obtained samples were analyzed by LC-MS, which detected ~17,000 unique compounds. LC-MS measurements were performed with a quadrupole Orbitrap mass spectrometer (Q Exactive Plus, Thermo Fisher Scientific), operating in negative ion mode, and coupled to hydrophilic interaction chromatography via electrospray ionization. The scan range was *m/z* 73–1000.

### Chromatographic NADP(H) 2′-dephosphorylation assays

Analyses of NADP(H) cleavage were carried out at 22 °C using 1000 μM NADP(H) (Roche) and 0.5 μM Nocturnin, unless otherwise noted. Reactions contained 20 mM Tris·HCl (pH 8.0), 70 mM NaCl, and 2 mM MgCl_2_. The reactions were stopped at indicated times by quick heating to 95 °C. Samples were injected onto a Mono-Q column on AKTA fast performance liquid chromatography instrument (GE Healthcare Life Sciences) and eluted in 20 mM Tris·HCl (pH 8.0) with 0→2 M NaCl gradient. Standards for NAD^+^, NADH, NADP^+^, and NADPH were from Roche. Peak areas were used to quantify metabolite abundances and determine cleavage kinetics.

### Radiolabeled RNA cleavage

RNA oligonucleotides were purchased from Integrated DNA Technologies. 2 pmol of nucleic acid were 5′ radiolabeled with T4 polynucleotide kinase (New England BioLabs) and γ-32P ATP (Perkin Elmer) in 1 × T4 polynucleotide kinase buffer for 30 min at 37 °C. The substrates were resolved on a denaturing polyacrylamide gel, visualized by autoradiography, excised from gel, and placed in a 0.3-mL solution of 0.3 M sodium acetate overnight at 4 °C followed by ethanol precipitation and resuspension in sterile water.

Kinetics analyses with radiolabeled nucleic acid substrates were carried out at 22 °C using the concentrations of 2 nM nucleic acid and 2 μM enzyme. Reactions contained 5 mM HEPES pH 7.5, 70 mM KCl, 2 mM MgCl_2_, 5% (vol/vol) glycerol, 1 mM DTT, 150 μM Spermidine, and 0.02% NP-40. The reactions were stopped at indicated times with the addition of quencher dye (90% formamide, 2.5% glycerol, 0.01% SDS, 0.01% bromophenol blue, 0.01% xylene cyanol, 1 mM EDTA) and heated for 10 min at 95 °C. The samples were then run on 20% polyacrylamide denaturing gels and visualized by phosphorimaging.

### Immuno-precipitation of human FLAG-NOCT from A549 cells

Human A549 cells grown in 10 cm dishes were transfected with 8 μg pcDNA^TM^4/TO plasmids expressing either full-length WT or E195A human NOCT, with a C-terminal FLAG tag. Transfections were carried out using Lipofectamine 2000 (Invitrogen) per manufacturer′s instructions, for 18 h. Cells were washed once, scraped in cold PBS, transferred to 1.5 ml Eppendorf tubes and pelleted at 500 × *g* for 5 min (at 4 °C). The cell pellets were resuspended in lysis buffer (10 mM HEPES pH 7.5, 10 mM NaCl, 2 mM EDTA and 0.5% Triton X-100), supplemented with complete protease inhibitor cocktail (Roche), RNase inhibitor, then constantly rotated at 4 °C for 10 min. Cell lysates were centrifuged at 1000 × *g* for 10 min at 4 °C to pellet nuclei. Supernatants were incubated with anti-FLAG M2 magnetic meads (Sigma; the list of antibodies used in the work is provided in Supplementary Table 3), which were pre-equilibrated with lysis buffer. After incubating beads with cell lysates at 4 °C for two hours, beads were washed three times for 2 min in ice-cold wash buffer (10 mM HEPES pH 7.5, 150 mM NaCl, 0.5% Triton X-100). Lastly, beads were washed and resuspended in reaction buffer (20 mM Tris·HCl pH 8.0, 70 mM NaCl and 2 mM MgCl_2_), and immediately used for assays. Beads incubated with naïve A549 cells lysates served as a control for non-specific interactions.

### Microscopy

A549 cells were seeded on a chamber slide (Lab-Tek) in RPMI-1640 medium (Sigma) supplemented with 10% FBS at 37 °C. Cells were transfected using Lipofectamine 2000 (Invitrogen) with pcDNA^TM^4/TO plasmids (Life Technologies) encoding either WT full-length human NOCT, E195A full-length human NOCT or ΔMTS human NOCT. Twenty four hours after transfection, cells were briefly washed with serum-free RPMI-1640 and stained with 5 μM MitoSOX™ Red (Invitrogen, Lot #1985406) at 37 °C for 10 min. Cells were washed again with PBS, fixed with 4% paraformaldehyde in PBS at room temperature (15 min), permeabilized with 0.1% TX-100 in PBS (10 min), then blocked with 20% goat serum in PBS (20 min). Mouse anti-FLAG (1:500, M2 Sigma) was used as a primary antibody and AlexaFluor 488 goat anti-mouse (1:500, Life Technologies, Lot #1613346) was used as a secondary antibody. Cells were further stained with DAPI (1:1000, Thermo Scientific, Lot #PJ1919631) and images were taken with Nikon Instruments A1 Confocal Laser Microscope. Nikon Elements software was used to prepare the images.

### Subcellular fractionations and western blots

The fractionation protocol was adapted from Jean Beltran et al.^29^. Three 15 cm dishes with A549 cells were transfected using Lipofectamine 2000 (Invitrogen) with plasmids expressing human NOCT (WT, E195A or ΔMTS). Cells were harvested 24 h post-transfection pelleted at 4 °C and resuspended in lysis buffer (10 mM HEPES (pH 7.4), 10 mM NaCl, 2.5 mM MgCl_2_). The lysates were supplemented with complete protease inhibitor cocktail (Roche) and placed on a rotator for 10 min. Resuspended cells were further disrupted with 20 strokes on a dunce homogenizer. Lysate were centrifuged at 700 × *g* for 10 min to pellet nuclei, which were kept for further processing. The supernatant was centrifuged at 20,000 × *g* for 30 min to pellet organelles, including mitochondria. The resulting supernatant, containing the cytosolic fraction, was kept for further processing.

The organelle pellet was resuspended in 0.25 M sucrose, 6 mM EDTA, 120 mM HEPES pH 7.4, and top-loaded in a discontinuous density gradient established by iodixanol (Sigma OptiPrep^TM^) at 0, 10, 15, 20, 25, and 30%. The tubes were centrifuged at 130,000 × *g* for three hours using an SW 60-Ti rotor (Beckman). Fractions from the 15 and 20% layers were diluted with PBS then centrifuged at 20,000 × *g* for 30 min to pellet the mitochondria. The nuclear pellet and the mitochondrial pellet were resuspended in Tris-buffered saline and lysed by sonication. Total protein concentration in each fraction was determined by Bradford assay. Equal OD_595_ units were loaded and separated by 10% BisTris PAGE (NuPAGE). The gels were transferred to PVDF membranes (Life Technologies), followed by 15 min of blocking with 5% milk. The membranes were incubated with 1:1000 mouse anti-human PARP-1 antibody (Santa Cruz Biotech), 1:1000 mouse anti-human COX4 antibody (Santa Cruz Biotech), 1:1000 mouse anti-human α1a Tubulin antibody (Santa Cruz Biotech), or 1:1000 mouse anti FLAG (Sigma) primary antibody at 4 °C, overnight. The membranes were washed with Tris-buffered saline-Tween buffer and incubated with horseradish peroxidase-conjugated with anti-mouse secondary antibody (1:10,000, Jackson ImmunoResearch) for 30 min. The membranes were washed again and visualized using X-ray film and enhanced chemiluminescence western blotting detection reagents (GE Healthcare Life Sciences).

### Construction of A459 NOCT-KO cells

The oligonucleotide sequences used for the generation of small guide RNAs (sgRNA) are shown in Supplementary Table 2. A pair of forward and reverse oligonucleotides for the generation of each sgRNA (synthesized by IDT) was annealed and inserted into plasmid vectors pLenti-CRISPRv2 (a gift Prof. Alex Ploss, Princeton University), between BsmBI restriction sites. For lentivirus production, HEK293T cells were plated into six-well plates to achieve 50–60% confluency in 24 h. On the next day, cells were transfected with 1.5 μg pLenti-CRIPSRv2 (with sgRNA), 1.33 μg pCMVdR8.91, and 0.17 μg of pMD2.G (a gift from Prof. Jared Toettcher, Princeton University), using FuGENE HD Transfection reagent (Promega) (9 μL in 150 μL of OptiMEM per well). Lentivirus-containing medium was collected after 48 h. Following collection, the medium was passed through 0.45 μm filter. Polybrene 5 μg/mL (f/c) and HEPES (pH 7.5; 100 mM f/c) were then added.

A549 cells at 40% confluency were infected with 100 μl of lentivirus-containing media in six-well plates. The media was changed 24 h post-infection, and puromycin (2 μg/ml f/c) was added three days post-infection. After four days of selection, surviving cells were cloned by limited dilution. Single-cell clones were selected for further amplification and genotyping and, independently, transcript-level mutation confirmation.

### RNA-seq

Poly-A^+^ RNA-seq samples from human A549 cell line were generated, sequenced and mapped to hg19 human genome assembly as previously described^30,31^.

### GSEA analysis

Gene set enrichment analysis was conducted in GSEA (Broad Institute) as previously described^31^. The output enrichment curves for highly significant terms are shown. False discovery rate (FDR) provides a statistically weighted significance corrected for background and computed via multiple hypotheses testing routine. GSEA FDR ≤0.25 is significant. All enriched terms shown on our figures and related to mitochondria have FDR <10^−6^.

### NOCT•NADPH co-crystallization

Crystallization drops contained 12 mg/ml of NOCT 122–431, 2 mM CaCl_2_, and 100 mM NADPH (Roche). Calcium was selected instead of magnesium to stabilize the metabolite. Our experiments determined that NOCT is inactive in the presence of Zn^2+^ and Ca^2+^ presumably due imperfect transition state formed due to a slight size mismatch of these ions. Co-crystals were grown using the hanging drop vapor diffusion method by mixing the crystallization complex 1:1 with reservoir solution (100 mM HEPES pH 7.5, 10% (v/v) Isopropanol, 50 mM sodium acetate, and 12% (w/v) PEG 4000). Crystals were cryoprotected using 100 mM HEPES pH 7.5, 10% isopropanol, 50 mM sodium acetate, 12% (w/v) PEG 4000, 100 mM NADPH, 2 mM CaCl_2_, and 25% (v/v) glycerol, then frozen in liquid nitrogen.

### X-ray data collection and structure determination

X-ray diffraction data were collected at the AMX (17-ID-1) beam line at the Brookhaven National Laboratories (NSLS-II). Data were collected at a wavelength of 0.97 Å and processed with XDS and then STARANISO to correct for diffraction anisotropy. Co-crystals contained a single NOCT copy per asymmetric unit and belonged to the tetragonal P4_1_2_1_2 space group. The structure was solved by molecular replacement in PHASER, using the human apo NOCT structure (PDB ID 6MAL) as the search model. Structure of the complex was built in COOT and refined using simulated annealing (5000 K, then 2000 K for final stages) using PHENIX.

### X-ray structure visualization and analysis

Structure images were generated using PyMol (DeLano Scientific built) and UCSF Chimera^32^. Sequence conservation and 3D conservation mapping was conducted using SEQMOL-Kd (BiochemLabSolutions.com).

## Supplementary information

Supplementary Information

Peer Review File

Reporting Summary

Description of Additional Supplementary Files

Supplementary Data 1

Supplementary Data 2

Supplementary Data 3

Source Data

## Data Availability

RNA-seq data have been submitted to GEO database under ID GSE123477. Mass spectrometry data were deposited to Peptide Atlas under accession ID PASS01369. Structure and the diffraction data have been submitted to PDB database under ID 6NF0. The source data underlying Figs. 1b, 2f, 3e, f and Supplementary Fig. 3C–D are provided as Supplementary Data 1–3. Source data for Figs. 2c, d, f, 3b, c, 5b, c and Supplementary Figs. 2a, c and 5a, c are provided as Source Data.
