# Supplementary material for: The metabolites NADP+ and NADPH are the targets of the circadian protein Nocturnin (Curled)
Source: Nat Commun. 2019 May 30;10:2367. doi: 10.1038/s41467-019-10125-z (PMC6542800; doi:10.1038/s41467-019-10125-z)
Supplement: Supplementary file 1 — Supplementary Information [file 41467_2019_10125_MOESM1_ESM.pdf]

Supplementary Figures

A

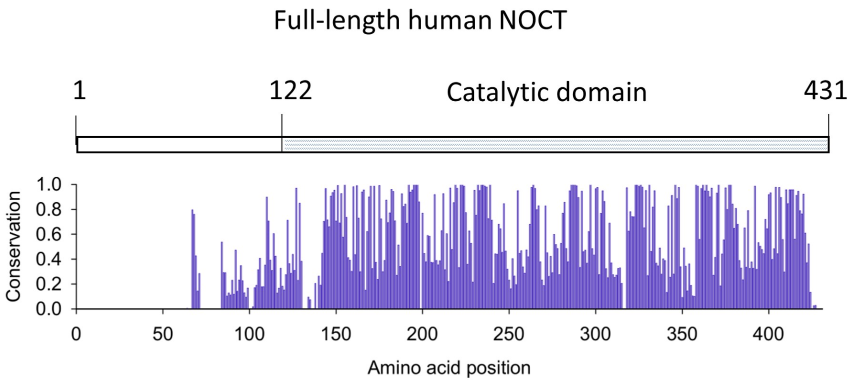

B

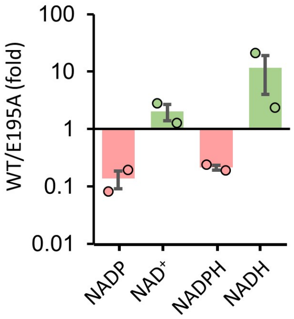

Supplementary Figure S1. NOCT sequence and metabolite conversion activity. A) Sequence alignment for 351 non-redundant NOCT sequences was used to compute the conservation values. Conservation was computed using SEQMOL-Kd (BiochemLabSolutions). **B)** The depletion of NADP(H) in the metabolite screen experiments (Fig. 1A) is accompanied by the expected increase in NAD(H). The basal levels of NAD^+ considerably exceed those of $NADP^+$, which accounts for a smaller magnitude of NAD^+ increase compared to $NADP^+$ loss. Error bars are standard error (S.E.).

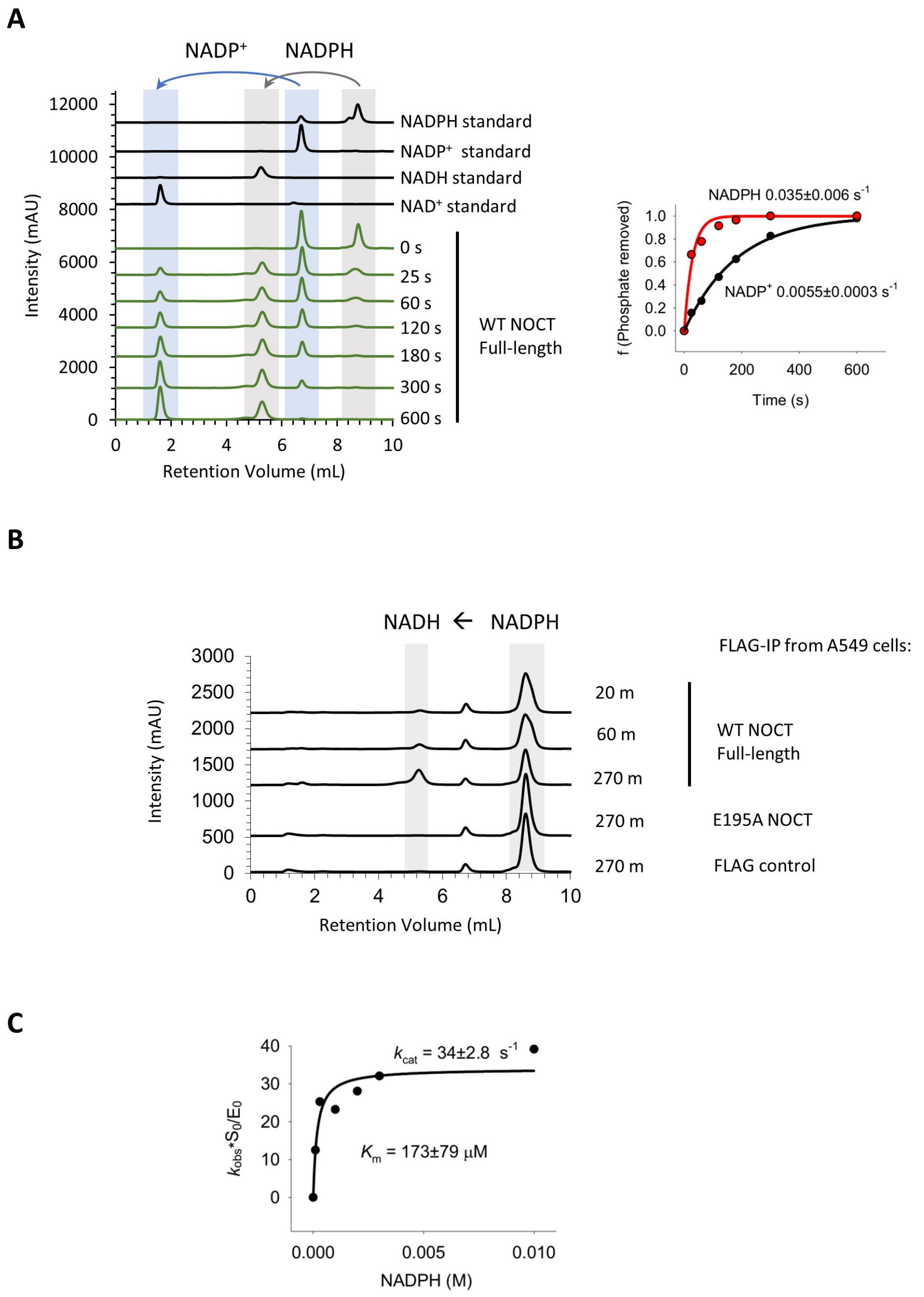

Supplementary Figure S2. NADP$^+$ and NADPH cleavage analyses. A) Left: Cleavage of NADP$^+$ and NADPH under conditions of kinetic competition. Reactions contained 0.5 μM full-length WT human NOCT and 1 mM of each metabolite. Right: quantitation of the data fitted to single-exponential kinetics. **B**) A biologic replicate of NADPH (1 mM) cleavage by full-length NOCT on beads, which was purified from

human cells (related to Fig. 2E). **C**) Kinetic parameters for NADPH cleavage by full length NOCT determined using 0.5 μM NOCT and as in Fig. 2C. Source data are provided as a Source Data file.

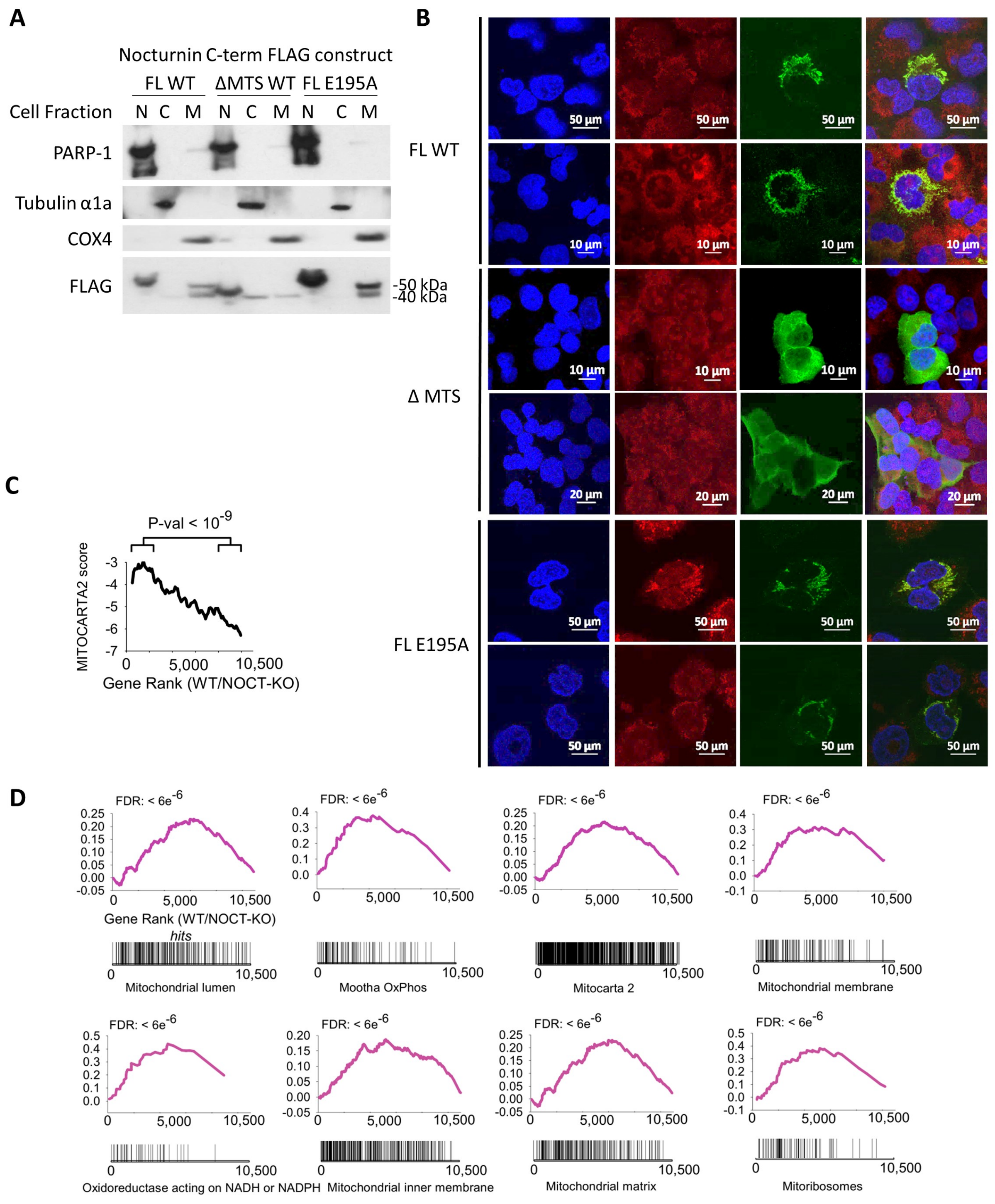

Supplementary Figure S3. NOCT localization and effect on transcription in human cells. A) Subcellular fractionation and western blot analysis of NOCT variants related to Fig. 3C. **B**) Confocal microscopy of FLAG-NOCT, WT, ΔMTS and E195A active site

mutant related to Fig. 3D. **C**) Running average of MitoCarta 2 scores for mRNAs upregulated in WT vs NOCT$^{-/-}$ cells, as determined by RNA-seq. The P-values were obtained as in Fig. 3E. RNA-seq data represent four RNA-seq experiments: two in WT cells and two in independently derived NOCT-KO clones. **D**) GSEA enrichment for mitochondria-related terms in WT vs NOCT$^{-/-}$ cells as in Fig. 3F. Source data are provided as a Source Data file.

A

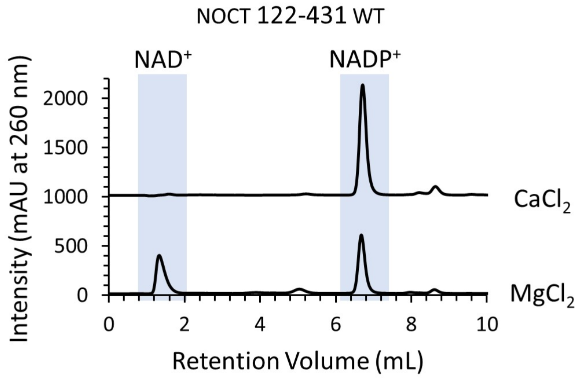

NOCT 122-431 WT
NAD+
NADP+
Intensity (mAU at 260 nm)
2000
1500
1000
500
0
CaCl2
MgCl2
0
2
4
6
8
10
Retention Volume (mL)

B

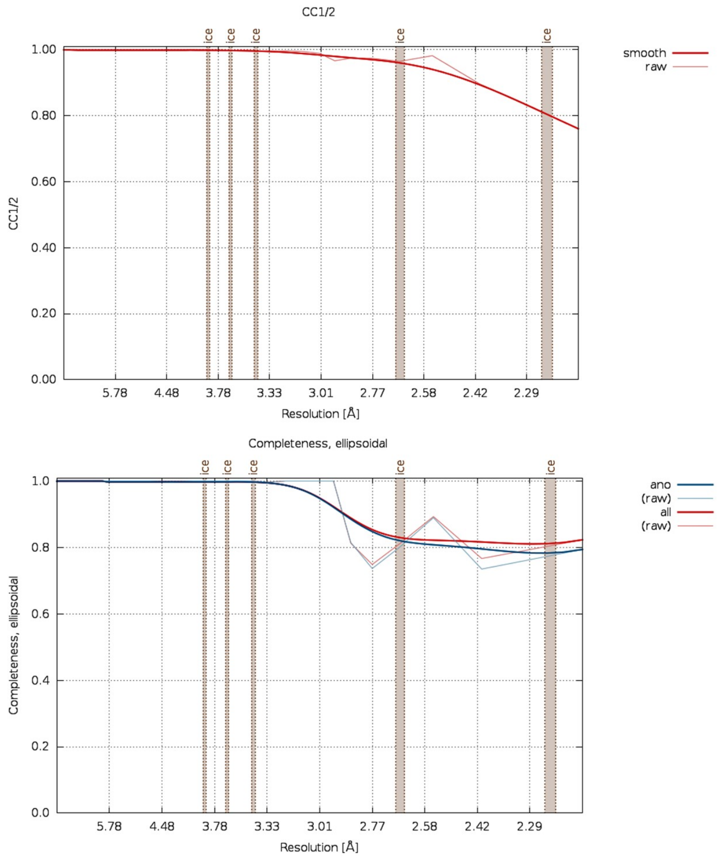

CC1/2
ice
smooth
raw
1.00
0.80
0.60
0.40
0.20
0.00
5.78
4.48
3.78
3.33
3.01
2.77
2.58
2.42
2.29
Resolution [Å]
Completeness, ellipsoidal
ano
(raw)
all
(raw)
1.0
0.8
0.6
0.4
0.2
0.0
Resolution [Å]

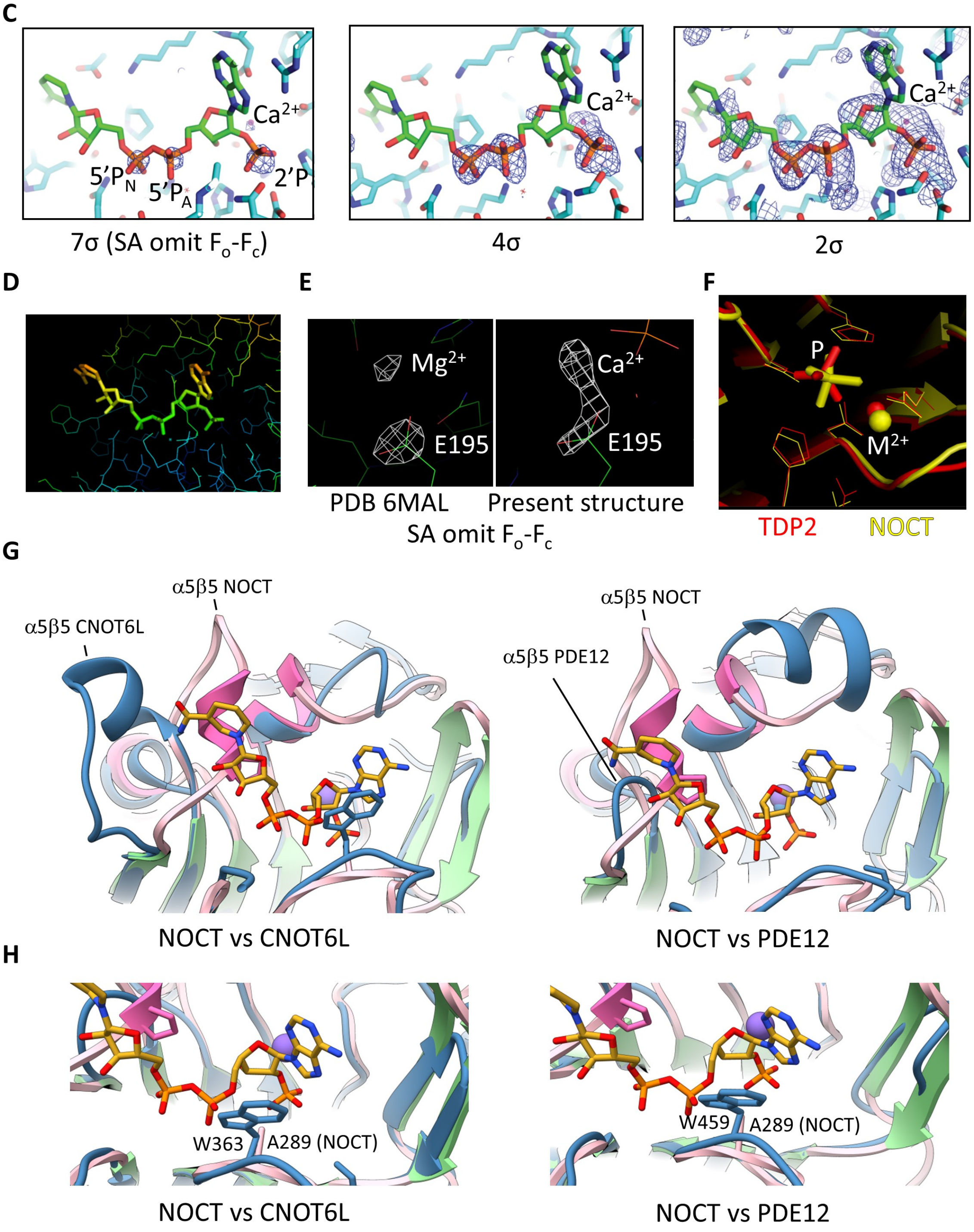

Supplementary Figure S4. Diffraction properties of NOCT•NADPH crystals and structure comparisons with deadenylases. A) NOCT 122-431 activity is impaired by the addition of 2 mM $CaCl_2$. Reactions were conducted for 30 minutes as in Fig. 2B. **B**)

Completeness and $CC_{1/2}$ plots for anisotropic diffraction data processing by STARANISO (http://staraniso.globalphasing.org/cgi-bin/staraniso.cgi). **C**) Simulated annealing (2000K) omit map for NADPH at three contour levels indicated on the figure. **D**) B-factor-colored NADPH (see also Supplementary Table S1). B-factors are scaled from highest (95.29, red) to lowest (21.91, blue). **E**) Simulated annealing omit map for the previously published NOCT•Mg^{2+} complex 6MAL (Mg^{2+} and CD+OE1+OE2 atoms of E195 were omitted) vs a similar map for the NOCT•NADPH•Ca^{2+} complex (Ca^{2+} and CD+OE1+OE2 atoms of E195 were omitted). The electron density for Ca^{2+} is stronger than that for Mg^{2+}, as expected due to higher electron count in Ca^{2+}. **F**) Superposition of the TDP2 DNA repair enzyme in complex with 5'-P DNA (PDB ID 5INL) vs the NOCT•NADPH•Ca^{2+} complex. **G**) NOCT superimposition with CNOT6L and PDE12 showing α5β5 motifs. **H**) NOCT superimposition with CNOT6L and PDE12 showing a tryptophan in place of the NOCT alanine 289.

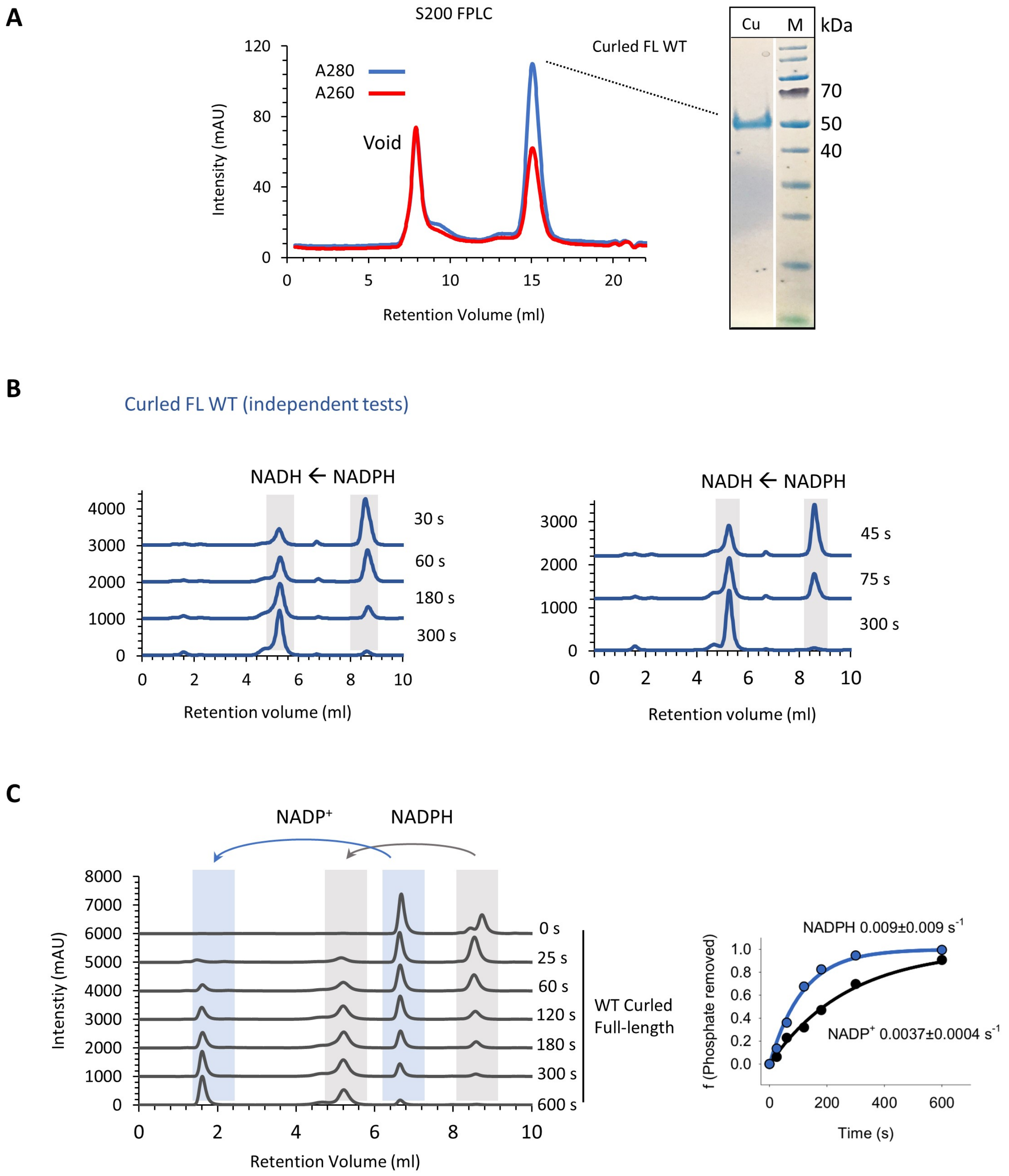

Supplementary Figure S5. Curled purification and activity assay. A) S200 size exclusion purification trace for full length *Drosophila melanogaster* Curled protein and NuPAGE analysis of the peak. The protein was concentrated to 0.9 mg/ml (19.23 µM).

B) Cleavage of NADPH (1 mM) by Curled (0.5 μM) related to Fig. 5C. **C**) Left: $NADP^+$ and NADPH cleavage by Curled under conditions of kinetic competition as in Supplementary Figure S2A. Reactions contained 500 nM Curled and 1 mM of each nucleotide. Right: quantitation of the data fitted to single-exponential kinetics. Source data are provided as a Source Data file.

Supplementary Tables

Supplementary Table S1. Data collection and refinement statistics[&]

	Human Nocturnin 122-431 • NADPH • Ca^{2+}
**Data collection**	
Space Group	$P4_12_12$
Cell dimensions	
a, b, c (Å)	62.88, 62.88, 153.59
α, β, γ (°)	90.0, 90.0, 90.0
Resolution (Å)	29.09-2.05 (2.33–2.05)
R_{pim}	0.034 (0.443)
CC(1/2)	0.999 (0.937)
$I/\sigma I$	18.04 (2.93) {14.27 (1.86)}*
Completeness	0.999 (0.998) {0.932 (0.824)}*
Redundancy	12.73 (12.06)
**Refinement**	
Resolution (Å)	2.70
No. unique reflections	8,470 (523)
R_{work}/R_{free}	0.2063/0.2630
No. atoms	2,429
Protein	2,362
NADPH	48
Ca	1
Water	18
B-factors	
Protein	48.67
NADPH	73.86
Ca	50.79
Water	40.44
R.m.s deviations	
Bond lengths (Å)	0.005
Bond angles (°)	0.802
Ramachandran plot (%)	
Favored	97.28
Allowed	2.72
Disallowed	0.0

Highest-resolution shell is shown in parentheses.
[&]See Source Data file for stereo map.
*Values from XDS vs {anisotropy processed values from staraniso}.

Supplementary Table S2. Oligonucleotide sequences

ID	Sequence 5'-3'	Use
ME66.hNOCT.Fwd	GTGTCTCGAGATGTTT<br>CATAGTCCGCGG	amplify from A549 cDNA
ME70.hNOCT.Rev	GTGTGCGGCCGCTTATGAAAGT<br>CCATCAGATTC	
ME68.hNOCT.FL.InFrame	CGGATCTGGAAGTTCTGTTCCAGG<br>GGCCCATGTTTCATAGTCCGCGGCGGCTCTGCTCG	NOCT in frame with pGEX HRV 3C
ME79.Noct.d1-121	CGGATCTGGAAGTTCTGTTCCAGGGG<br>CCCCCTCCCCGGTTCCAGAGGGATTTTGTGGATC	generate NOCT 122-431
ME81.Noct.E195A	CAGCCTGATATATTGTGCCTCCAA<br>GCTGTGGACCACTATTTTGACACCTTCC	point mutant
ME82.Noct.R290A	CATCGCTGTTACCCATCTAAAAGCAGCTACTGG<br>CTGGGAGCGGTTTCGATCAGC	point mutant
ME89.Noct.K365A.R367A	GTCAGAACCCCCATACACTACCTGGGCGAT<br>CGCGACCTCAGGGGAGTGCAGGCACACCC	point mutant
JD88.Noct.pGEX.N-FLAG	ctggaagttctgttccaggggCCCgactacaaggacgac<br>gatgacaagATGTTTCATAGTCCGCGGCGGCTC	add N-term FLAG to NOCT in pGEX
JD89.Noct.del.d1-125	gatctggaagttctgttccaggggCCCCAGAGGGATTTTG<br>TGGATCTGAGG	generate NOCT 126-431
JD90.Noct.R295A	ACGCACTGGCTGGGAGGCGTTTCGAT<br>CAGCTCAAGGC	point mutant
JD102.NOCT.D160A	AAGCTCTTGGAGAAGGCAAAGCGAACTTTGT<br>ACAGTGCCCTGTTGAAGC	point mutant
JD112.Noct.del.P220-N231	GGCTATCAAGGCACGTTTTTCCCCAAAGGACC<br>AGATGGTTGTGCCTTATTTTTTC	internal deletion
JD113.NOCT.D160A.F162Y	AAGCTCTTGGAGAAGGCAAAGCGAACT<br>ATGTACAGTGCCCTGTTGAAGC	point mutant
JD114.NOCT.R367A	CACTACCTGGAAGATCGCGACCTCAGG<br>GGAGTGCAG	point mutant
JD133.NOCT H286N	GTTCTGCATCGCTGTTACCAACCTAAAAG<br>CACGCACTGGCTG	point mutant
JD146.NOCT.D128A	CCTCCCCGGTTCCAGAGGGCGTTTGTGG<br>ATCTGAGGACAGATT	point mutant
SR.FlagNOCT-F_EcoRI	TATATAGAATTCCACCATGGATTACAAGGATGACG<br>ACGATAAGATGTTTCATAGTCCGCGGCGGCTC	subclone into pcDNA 4.0/TO
SR.NOCT-R_NotI	TATATAGCGGCCGCTTATGAAA<br>GTCCATCAGATTCCTC	
JD130.NOCT.loopout-MT	GATTACAAGGATGACGACGATAAGTATA<br>GTGCTCTCGCCAAGACACTGAACAGC	generate NOCT 75-431
ME92.N.FLAGdel.NOCT	AGTCCAGTGTGGTGGAATTCCACCATGGCGTTTC<br>ATAGTCCGCGGCGGCTCTGCTCGGCC	remove N-term FLAG tag
JD136.C.FLAGins.NOCT	TTTACTGAGGAATCTGATGGACTTTCAGATTACA<br>AGGATGACGACGATAAGTAAGCGGC<br>CGCTCGAGTCTAGAGG	adds C-term FLAG tag

JD95.PDE12-F2-EcoRI	CACAGAATTCCGGAAGTCGCGAGATCTGAA	amplify PDE12 from A549 cDNA
JD96.PDE12-R2-XmaI	CACACCCGGGAGGAGGGCTGAACATTTTACCA	
JD110.PDE12 d1-154	TCTGGAAGTTCTGTTCCAGGGGCCCTACAAGGT GGAGCGCAACCCGCCCGC	PDE12 155-609
JD99.CNOT6L-F-XhoI	ATATCTCGAGGGACCGAGAGTGTTGGGAAG	amplify CNOT6L from A549 cDNA
JD100.CNOT6L-R-NotI	ATATGCGGCCGCAGCAACAGATCCCCGTCTTG	
JD111.CNOT6L d1-157	ctggaagttctgttccaggggCCCATGCTTGACAATCT CGCAGTTCATCC	CNOT6L 158-555
ME95.Curled.inframe	CGGATCTGGAAGTTCTGTTCCAGGGGCCCATGGGTT CATTTAACTCGGCCCCAAAG	Curled in frame with pGEX HRV 3C
ME96.Curled.E140A	GAACCAGCCCGATGTGATTTGTCTGCAAGCGGTAGA CCACTTCAAGTTTCTGCAGACCG	point mutant
TA.NotI-cu-03	CTCGAGAATGGGTTCATTTAACTCGGCCCC	amplify Curled
TA.XhoI-cu-06	GCGGCCGCTTTCTATTGAATGGATCCATGC	
cu02	AGCTGCAAACTGTAGTGCAACG	reverse transcribe
Noct_sg3a	caccgCATTGCATAACCCTGATAGG	gRNAs for making NOCT CRISPR knockout
Noct_sg3b	aaacCCTATCAGGGTTATGCAATGc	

Supplementary Table S3. Antibodies used

Antibodies	Dilution Used	Manufacturer	Catalogue #	Lot #	Note
α1a Tubulin (7-RY28)	WB- 1:500	Santa Cruz Biotechnology	sc-134237	C1218	Raised in mouse
PARP1 (B-10)	WB- 1:500	Santa Cruz Biotechnology	sc-74470	B2118	Raised in mouse
COX4 (F-8)	WB- 1:500	Santa Cruz Biotechnology	sc-376731	D0418	Raised in mouse
Monoclonal ANTI-FLAG M2	IF- 1:500<br>WB- 1:2,000	Sigma	F3165	SLBT6752	Raised in mouse
Peroxidase-conjugated AffiniPure Goat Anti-Mouse IgG (H+L)	WB- 1:10,000	Jackson ImmunoResearch	115-035-062	132504	Raised in goat

Note: IF - immune-fluorescence, WB - western blot
