# Supplementary material for: The metabolites NADP+ and NADPH are the targets of the circadian protein Nocturnin (Curled)
Source: Nat Commun. 2019 May 30;10:2367. doi: 10.1038/s41467-019-10125-z (PMC6542800; doi:10.1038/s41467-019-10125-z)
Supplement: Supplementary file 3 — Reporting Summary [file 41467_2019_10125_MOESM3_ESM.pdf]

nature research

Corresponding author(s): Alexei Korennykh

Last updated by author(s): Dec 18, 2018

Reporting Summary

Nature Research wishes to improve the reproducibility of the work that we publish. This form provides structure for consistency and transparency in reporting. For further information on Nature Research policies, see Authors & Referees and the Editorial Policy Checklist.

Statistics

For all statistical analyses, confirm that the following items are present in the figure legend, table legend, main text, or Methods section.

n/a	Confirmed	
☐	☒	The exact sample size (n) for each experimental group/condition, given as a discrete number and unit of measurement
☐	☒	A statement on whether measurements were taken from distinct samples or whether the same sample was measured repeatedly
☐	☒	The statistical test(s) used AND whether they are one- or two-sided<br>*Only common tests should be described solely by name; describe more complex techniques in the Methods section.*
☒	☐	A description of all covariates tested
☒	☐	A description of any assumptions or corrections, such as tests of normality and adjustment for multiple comparisons
☒	☐	A full description of the statistical parameters including central tendency (e.g. means) or other basic estimates (e.g. regression coefficient) AND variation (e.g. standard deviation) or associated estimates of uncertainty (e.g. confidence intervals)
☒	☐	For null hypothesis testing, the test statistic (e.g. F, t, r) with confidence intervals, effect sizes, degrees of freedom and P value noted<br>*Give P values as exact values whenever suitable.*
☒	☐	For Bayesian analysis, information on the choice of priors and Markov chain Monte Carlo settings
☒	☐	For hierarchical and complex designs, identification of the appropriate level for tests and full reporting of outcomes
☒	☐	Estimates of effect sizes (e.g. Cohen's d, Pearson's r), indicating how they were calculated

Our web collection on statistics for biologists contains articles on many of the points above.

Software and code

Policy information about availability of computer code

Data collection	LC-MS, RNA-seq and X-ray data collection used standard procedures as described in methods.
Data analysis	All data analysis steps and programs were standard, published before and are described in methods.

For manuscripts utilizing custom algorithms or software that are central to the research but not yet described in published literature, software must be made available to editors/reviewers. We strongly encourage code deposition in a community repository (e.g. GitHub). See the Nature Research guidelines for submitting code & software for further information.

Data

Policy information about availability of data

All manuscripts must include a data availability statement. This statement should provide the following information, where applicable:

- Accession codes, unique identifiers, or web links for publicly available datasets
- A list of figures that have associated raw data
- A description of any restrictions on data availability

All data and accession codes are provided with the manuscript, in PDB database and in GEO database.

Field-specific reporting

Please select the one below that is the best fit for your research. If you are not sure, read the appropriate sections before making your selection.

☒ Life sciences ☐ Behavioural & social sciences ☐ Ecological, evolutionary & environmental sciences

For a reference copy of the document with all sections, see nature.com/documents/nr-reporting-summary-flat.pdf

Life sciences study design

All studies must disclose on these points even when the disclosure is negative.

Sample size	Number of replicates was selected to enure that variations are small compared to the reported effects. Our central findings are all-or-nothing (i.e. obtaining the structure, observing NADPH cleavage by WT but not mutant NOCT, and WT but not mutant Curled), which renders the key findings independent of technical variability.<br><br>For LC-MS, three independent biological tests identified NADP+/NADPH and deemed an adequate screen outcome. These results were confirmed by a highly redundant set of follow-up direct biochemistry tests and structural biology. NADP(H) cleavage was reproduced multiple times for both NOCT and Curled. Microscopy and fractionation gave the same results on different days.
Data exclusions	1. LC-MS: Fold ratios in Figure 1B were obtained after removing metabolites that have ≥20,000 counts in WT NOCT sample (true substrates should be completely depleted) and metabolites that have ≤10,000 counts in the input (i.e. these metabolites are not present in all controls).<br>2. Structure solving: standard data exclusion steps were used during X-ray data analysis, such as test set selection (5%), selection of the resolution cutoff. Table S1 provides statistics to assess the final data and the model quality.
Replication	LC-MS, RNA-seq, microscopy, fractionation, NADPH cleavage reactions and RNA cleavage by Curled were replicated and reproducible. We provide n, S.E. and P-values where appropriate.
Randomization	Not involved
Blinding	Not involved

Reporting for specific materials, systems and methods

We require information from authors about some types of materials, experimental systems and methods used in many studies. Here, indicate whether each material, system or method listed is relevant to your study. If you are not sure if a list item applies to your research, read the appropriate section before selecting a response.

Materials & experimental systems

n/a	Involved in the study	
☐	☒	Antibodies
☐	☒	Eukaryotic cell lines
☒	☐	Palaeontology
☒	☐	Animals and other organisms
☒	☐	Human research participants
☒	☐	Clinical data

Methods

n/a	Involved in the study	
☒	☐	ChIP-seq
☒	☐	Flow cytometry
☒	☐	MRI-based neuroimaging

Antibodies

Antibodies used	The antibody information is provided in Table S3
Validation	All antibodies were standard from commercial vendors. Correct gel migration of several overexpressed FLAG-tagged proteins was used to validate the key FLAG antibody.

Eukaryotic cell lines

Policy information about cell lines

Cell line source(s)	Prof. Susan Weiss's laboratory at UPenn as specified in methods
Authentication	Yes. Done by ATCC.
Mycoplasma contamination	Tested by Susan Weiss laboratory (the cell provider as specified in Methods), tests were negative
Commonly misidentified lines<br>(See ICLAC register)	*Name any commonly misidentified cell lines used in the study and provide a rationale for their use.*
