# Supplementary material for: The metabolites NADP+ and NADPH are the targets of the circadian protein Nocturnin (Curled)
Source: Nat Commun. 2019 May 30;10:2367. doi: 10.1038/s41467-019-10125-z (PMC6542800; doi:10.1038/s41467-019-10125-z)
Supplement: Supplementary file 4 — Description of Additional Supplementary Files [file 41467_2019_10125_MOESM4_ESM.pdf]

Description of Additional Supplementary Files

File Name: Supplementary Data 1
Description: Mass spectrometry metabolite screening data from bovine liver and HEK293 cells. Fold ratios (Fig. 1B) were obtained after removing metabolites that have ≥20,000 counts in WT NOCT sample (i.e. true substrates must be strongly depleted). Metabolites with ≤ 10,000 counts in the input (10,000 counts is the background) were removed to examine only robustly detected compounds found in all controls.

File Name: Supplementary Data 2
Description: Mass spectrometry metabolite data obtained in human A549 WT cells and two independent clones of $NOCT^{-/-}$ cells.

File Name: Supplementary Data 3
Description: $Poly\text{-}A^{+}$ RNA-seq read counts from A549 WT cells and two independent clones of $NOCT^{-/-}$ cells.
