# Supplementary material for: The metabolites NADP+ and NADPH are the targets of the circadian protein Nocturnin (Curled)
Source: Nat Commun. 2019 May 30;10:2367. doi: 10.1038/s41467-019-10125-z (PMC6542800; doi:10.1038/s41467-019-10125-z)
Supplement: Supplementary file 2 — Peer Review File [file 41467_2019_10125_MOESM2_ESM.pdf]

Editorial Note: This manuscript has been previously reviewed at another journal that is not operating a transparent peer review scheme. This document only contains reviewer comments and rebuttal letters for versions considered at Nature Communications. Mentions of prior referee reports have been redacted.

REVIEWERS' COMMENTS:

Reviewer #1 (Remarks to the Author):

As Reviewer 1 for the original submission, I definitively confirm that this is a relevant manuscript that clarifies a "classical" problem in Drosophila genetics and opens new avenues to the investigation of the link between human metabolism and the circadian clock. I find this revised version manuscript much improved. The authors have carefully addressed all comments made by the Reviewers. I think that the NADP(H) phosphorylase activity has been clearly demonstrated. Likewise, the data on the mitochrondrial localization appear convincing. With regard to the X-ray day, obviously the crystals are suboptimal because of their anisotropy. The electron density for NADP is not beautiful. Nevertheless, the authors critically analyze their X-ray data and support them by other studies. The structural comparisons provide clues about the reactivity of nocturnin relative to the structurally related RNA deaminases.

I think that this paper will surely impact the field.
